# Structural Predestination of Individual Diversity in the Course and Severity of Infectious Diseases

**DOI:** 10.1100/tsw.2002.94

**Published:** 2002-01-23

**Authors:** Sergey N. Rumyantsev

**Affiliations:** Institute of Vaccines and Sera, Saint Petersburg, Russia

**Keywords:** biodiversity, clinical immunology, heterozygosity, infection, severity of disease

## Abstract

Infectious diseases can be manifested by a spectrum of clinical signs and resultant clinical courses that range from acute to chronic and possible persistence in the victim in a latent form. Until recently, the origins of this kind of biodiversity were poorly understood, but advances in immunology — especially in identifying the constitutional mechanisms of immunity — have contributed to our understanding of the origins of biodiversity in infectious diseases. Infectious diseases affect only focal areas in the affected organisms, and the amounts and distribution of infectious lesions vary from patient to patient. In a population attacked by an infectious agent, individuals can be conveniently divided into three categories: totally resistant organisms which contain no susceptible structures and are not affected; mildly susceptible organisms in which a few foci appear and in which the infection runs a benign course; organisms in which the number of constitutionally susceptible structures is high and the infectious process develops in a severe form. The diversity is determined by the differences in susceptibility of various parts of the organism under consideration.

**DOMAINS:** biomathematics and statistical genetics, enzymology and protein - protein interaction, gene expression, genetic engineering, genetics (evolutionary), genetics (fly), genetics/genomics/proteomics, genetics (man), genetics (mouse), genetics (plants), genetics (worms), genetics (zebrafish), genomics, molecular and gene therapy, molecular biology, molecular engineering, molecular evolution, molecular ecology, pharmacogenomics, transcription and gene regulation, signaling, drug receptor, experimental medicine, molecular medicine, molecular pharmacology, pharmacology, vaccine discovery, cell and tissue culture, cell biology, differentiation and determination, aging, atherosclerosis, development, growth and growth factors, infection, metabolic disease, microbial pathogenesis, osteoporosis, pathology, trans membrane signaling, cardiovascular biology, gastroenterology, hematology, immunology microbiology, microbiology (bacteriology), microbiology (fungal biology), microbiology (parasitology), microbiology (virology), pulmonology, structural biology, tissue engineering, ecosystems and communities, ecosystems management

## VARIABILITY OF INFECTIOUS LESIONS

Every infectious disease affects specific areas in the infected organism with focal lesions of characteristic distribution. The focal lesion of cutis is seen, for instance, during poxvirus infection. It consists of a number of skin lesions scattered over the surface of the body, each of which is the seat of inflammation so intense as to lead to the formation of small abscesses in the course of 4 to 5 days (Fig. 1). Beyond the edge of the lesions, the skin is normal. The number of lesions present may be less than a dozen in a minor case of illness, or they may number in the thousands. In a severe course of disease, lesions may be set so closely as to conceal almost the whole cutaneous surface[1]. The smallpox rash is most severe on the face, then the hands and upper extremity. From the hands upwards, the scabs diminished in density. On the front part of the trunk they are scarce, especially on the abdomen. The areas adjacent to the specific damage are found almost intact, though all the skin is presumably homogeneous[1,2,3].

Poliovirus infection targets nerve cells at random although the virus is present throughout the nervous system[4]. The brain and spinal cord are involved in every case of poliomyelitis, but not every area of these organs is susceptible to the virus. This can only mean that nerve cells in certain areas are susceptible to viral damage, whereas those in other areas are not. In the worst cases, nearly every skeletal muscle may be paralysed. In the mildest cases, paralysis may be limited to part of one muscle. All degrees and combinations of paralyses can be observed[3].

Mosaic distribution of damages is also characteristic of hepatitis, tuberculosis, and many other infections that chiefly affect internal organs[3,5]. The major recognized forms of Q fever are pneumonia, hepatitis, meningoencephalitis, vascular infection, bone infection, osteomyelitis, endocarditis, unexplained fever, and flu-like form[6]. Typhoid and paratyphoid fever produce inflammatory foci in many organs and, in some areas, necrosis and sloughing of tissues. Necrotic or inflammatory foci are commonly seen in the spleen and the liver, and sometimes in the kidney, the meninges, the bones, the endocardium, the joints, and many other portions of the body[3].

In bacillary dysentery, the specific pathological changes are located in the colonic mucosa and consist of random patches of greyish membrane composed of thickened, necrotic mucosa. In severe cases, the disease may produce the destruction of the whole of the large bowel. In less severe cases, the areas of necrosis of the mucous membrane are less extensive, with small, scattered ulcers or nodular crusted lesions appearing on the bowel wall[3]. Focal bone lesions were characteristic of some infections in bears during the Pleistocene era, circa 10,000 years B.C.[7].

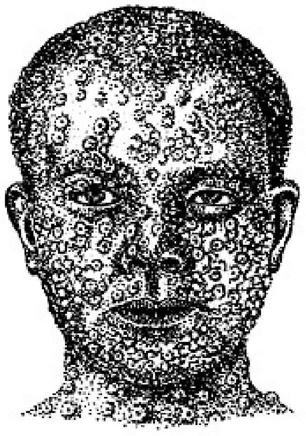

**FIGURE 1.** Mosaic focal distributions of specific lesions in a case of human smallpox.

## CYTOLOGICAL OBSERVATIONS

Heterogeneity of cell populations, which are sensitive to an infectious agent, has been reported frequently by many investigators. Sensitivity may involve only a few cells or the majority of cells in a population. When a susceptible cell culture is affected by some microbial pathogenic factor, most cells are killed. At the same time, however, a number of outwardly indistinguishable cells fully preserve their basic functions despite unfavourable conditions.

Cell strains that are spontaneously immune to the lethal effect of diphtheria toxin have been selectively isolated from highly sensitive human cell lines. The toxin-immune cells were found to be 100,000 to 1,000,000 times more resistant to toxin than the toxin-sensitive cells of the parent cell population[8,9]. Thus, the resistance of these cell strains was comparable to the resistance of cells from animal species that are naturally immune to the toxin, such as rat and mouse species. This phenomenon is highly specific: the cell may be immune to microbial agents of one type, but susceptible to another type.

According to Black-Schaffer et al.[10], the number and size of infectious lesions depend on the patient reactivity and the potency of the infecting strain. The examples considered above illustrate the manifestation of the mosaic configuration within affected homogenous structures. Evidently, the ubiquitous phagocytes and immunoglobulins are not functional in this phenomenon. The focal nature of infection is not explained by the lack of uniform predilection of micro-organisms for the organism. Every infection in its pre-eruptive stage is a septicaemia, and the causative agents are circulated everywhere in the affected tissue but they induce specific eruption only in susceptible areas of the tissue. In such cases, the infectious agent is found within both the typical damaged elements and the adjacent areas that have been left intact[2,3,11]. The mechanisms involved in the discussed phenomenon are of constitutional origin. Constitutional immunity explains why parts of one category are affected by a given microbe, while at the same time, morphologically identical components of the organism remain uninvolved although both exist under the same conditions and may be equally attacked by the agent.

### Constitutional Immunity

Immunity is the state of being immune; that is, having a degree of natural or acquired resistance to a disease[12]. Immunity involves an extremely complex set of surveillance systems that provide a remarkable group of agents, each geared to the maintenance of a healthy existence. Some of the systems are already well known.

Vertebrate animals possess several systems of immunogenesis (Fig. 2). Constitutional and phagocytic mechanisms serve as a first line of defense. If they are ineffective, the attacked organism can be rescued by the influence of specific immunoglobulins and other inducible agents that are produced mainly by the lymphatic system of reactive immunity.

Constitutional immunity is a general all-biological principle that provides all kinds of living organisms with inherent antimicrobial defence. It is not provided by immunoglobulins but by other mechanisms. They develop during ontogeny without induction by any particular parasite or parasitic product. These mechanisms were discovered just recently.

Just 35 years ago in his handbook on the fundamentals of immunology, W. Boyd[13] observed that constitutional immunity is a fundamental trait while expressing regret about the lack of scientific knowledge of this type of resistance. In the years since Boyd's comments, the situation has dramatically changed. Constitutional immunity in relation to many ecological and physiological effects has been revealed and, in many cases, its genetic determinants and molecular tools have been discovered[14,15,16,17,18,19].

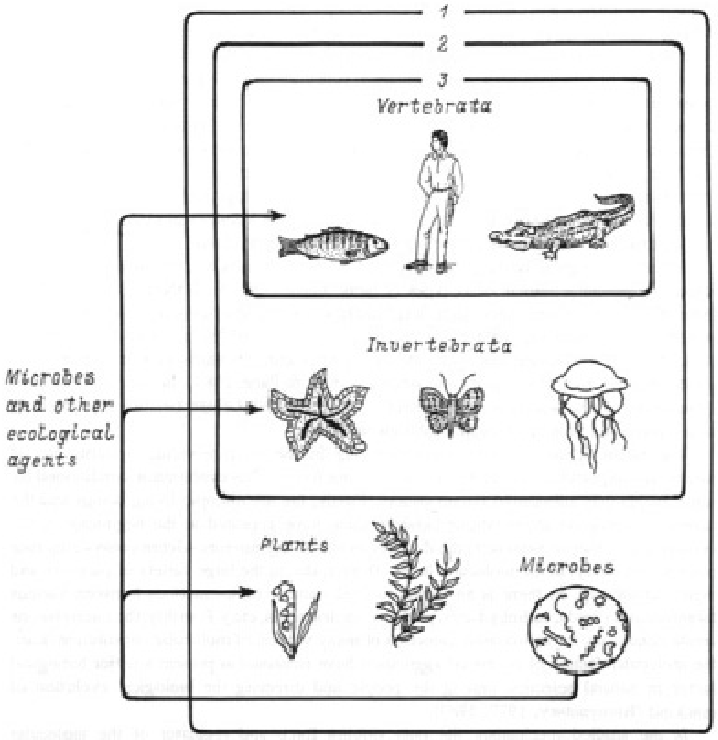

**FIGURE 2.** The main systems of immunogenesis: constitutional (1), phagocytic (2) and lymphatic (3).

## Immunity of Groups, Populations, and Species

Sheep, mice, guinea pigs, rabbits, and monkeys are susceptible to experimental anthrax, whereas dogs, horses, cats, rats, and pigs are resistant. Herbivores such as guinea pigs, rabbits, and horses are highly sensitive to all types of botulinic toxin. Of the carnivores, dogs and cats are highly immune to all types of botulinic toxin. Rhesus monkeys (*Macaca mulatta*) are resistant to type D botulism but sensitive to the other types[14]. Donkeys, horses, monkeys, and guinea pigs are very susceptible to tetanus, whereas hedgehogs, rabbits, dogs, cats, and chickens possess innate constitutional immunity to this pathogen[14,20]. Rats and mice, in contrast to monkeys, chickens, pigeons, and rabbits, can be affected by diphtheria toxin only after the exposure is increased to 50,000 times the amount that killed guinea pigs. Both rats and mice have natural immunity to diphtheria toxin, although there is no apparent evidence of specific immunoglobulins in their bodies[8].

Such natural immunity is not adequate in all representatives of a species. The rodent species *Meriones meridianus*, which lives on the left bank of the lower river Volga, is 70,000-fold less susceptible to the plague than representatives of the same species that live on the right bank of the same river. In regions of Africa where trypanosomiasis is endemic, indigenous ruminants suffer from mild infections with insignificant morbidity, whereas domestic animals suffer more severely, and recently imported exotic animals suffer usually fatal infection[21].

## Hereditary Nature

Genetic factors play a significant role in the incidence and severity of infectious diseases. In a study of an epidemic of tuberculosis in a herd of cattle descended from two bulls, 62% of the descendants of one bull were infected with tuberculosis whereas only 4% of the descendants of the other bull developed the disease[22]. Webster[23] was the first to develop the Mendelian ratios for the inheritance of immunity to microbial infections by mating constitutive immune and constitutive susceptible strains of mice. Through selective breeding, he developed strains of mice that differed in resistance to St. Louis and louping ill flaviviruses. He showed that immunity is inherited as a single, dominant factor.

Genes that control constitutional immunity to some infections in humans and animals have been identified and mapped to a specific chromosomal location[15,24,25,26,27]. The CCR5 gene 32-base pair deletion provides strong constitutional immunity of human homozygotes to HIV infection[28]; in the heterozygous state it may provide relative immunity, thus delaying the progression of HIV infection to AIDS in affected individuals[29].

## MOLECULAR MAKE-UP

Pathogenic microbes survive as a result of biosynthetic processes carried out by other organisms. This requires the ability to invade the victim, to penetrate its surfaces, to reproduce in the victim, to interfere with its defences, and to damage the victim. Microbes penetrate organisms and enter cells exclusively through particular molecular processes controlled by specific chemical (molecular ecological) substances (adhesins, toxins, enzymes, cytolysins, or polynucleotides). Infectious disease develops as a result of the interaction between the molecular ecological agents of pathogenic micro-organism on the one hand and the affected body molecular structures on the other. Specificity of host-parasite interaction involves mutual molecular recognition of host and parasite, mediated by chemically complementary molecules.

Harmful micro-organisms usually attack the animal host at the mucosal surfaces of the respiratory, gastrointestinal, or genitourinary tract. Micro-organisms must bind firmly to epithelial cells to avoid being swept away by the secretions that bathe the mucosal surface. In many of the common strategies used by pathogenic micro-organisms to overcome host defence, the first step is the firm adhesion of the micro-organism to the host cell membrane. Adhesion is mediated by glycoprotein molecules called adhesins. Adhesins are the molecular substances produced and used by microbes to adhere to target cells.

Bacterial viruses adhere to the surface of a victim by virtue of certain determinants, which, depending on the species of virus, may be derived from one to ten specific protein molecules. Protein components play a similar role in influenza infection: these are the so-called hemagglutinins, which function by binding to host cell sialosaccharide receptors. Some viruses, such as Sendai virus, have surface glycoprotein spikes that adsorb on specific receptors of the cells attacked by the virus. The receptors contain sialic acid.

Microbial adhesion is a highly specific phenomenon. The remarkable specificity exhibited during interaction of microbial adhesins and host cells has been compared with antibody/antigen specific recognition. A malaria-invasive molecular ecological agent, the 175-kDa erythrocyte-binding superficial protein molecule of *Plasmodium falciparum*, recognises the terminal Neu5Ac(alpha2-3)Gal-sequences of glycophorin A, a molecular component of the erythrocyte surface[30]. Recognition by malaria molecular ecological agents of sialic acid–containing receptors on the erythrocyte surface might be analogous to influenza virus entry into cells of the victim by means of hemagglutinin, which binds with stereospecificity to sialic acid–capped oligosaccharides. The adhesion of *Escherichia coli* to erythrocytes occurs if the cell surface contains glycophorin Am[31].

Choleraic toxin is able to interact only with ganglioside macromolecules that contain the subunits of ceramide, lactose, galactosamine, galactose, and the radical of sialic acid joined with the molecule of lactose. But the same toxin does not interact with gangliosides of other types; for instance, those that do not contain galactose or have an additional radical of sialic acid in the end position[32].

Thus, the pathogenic effects of microbes and microbial molecules on molecular targets of the victim are provided by the genetically determined molecular structure of the victim, thereby defining constitutional susceptibility to infection or invasion. As a result of this property of a molecular constitution, viability and propagation of intruders can be restricted or stopped. Constitutional antimicrobial immunity is a complex phenomenon, encompassing several specific and common structures possessed by the attacked organisms and used in defence against the molecular ecological aggression of harmful micro-organisms. These structures are presented with a variety of molecular barriers such as a lack of specific cell receptors, modification of the specific cell receptor, modification of specific nutrients, lack of a specific nutrient, presence of specific antibiotic and\or poison, and nonspecific mechanisms[19].

### Acquiring Through Selection

Certain modifications of an organism's molecular constitution can arise as a result of mutations. The mutations bringing about the origin of similar modifications of the molecular constitution of the victim reduce or even eliminate the efficacy of adequate intermolecular interactions and, accordingly, reduce the success of the microbial aggression. The organisms possessing this modified molecular constitution are incapable of interacting with the microbes; they are not susceptible to this disease and are constitutionally immune to it. Thus, these organisms are not affected by the corresponding microbe but, in contrast, continue to develop normally and produce healthy progeny while other individuals of the same species become ill and die. On repeated exposure to a given pathogen, the progeny of resistant mutants will finally predominate in a population; an individual protective mutation becomes the property of a group, then of a population, and finally of the species (Fig. 3).

Living beings have been subjected to interaction with agents of numerous infectious diseases due to their specific ecological features over millions of years. Species living today presumably possess genes for natural defence against the majority of those diseases[19]. As a consequence, constitutional resistance currently seems to be a leading factor in the immunological structure of the population determining the main parameters of epidemic processes including severity of diseases.

## ORIGIN OF THE GENETIC MOSAIC

The mating of resistant and susceptible individuals gives rise to progeny with intermediate degrees of infectious lesions; thus, hybrids can never be more resistant than their parents[2,33]. These genetically predetermined differences are proof of heterozygosity: the constitutionally immune parent passes on to the descendant a lack of receptivity to a certain infection in some parts of organs, while the susceptible parts are inherited from the second parent. There is no such thing as a hybrid between the two parents.

The clonal histochemical architecture of the small intestine, oesophagus, breast, and thyroid was demonstrated in normal animals using a diversity of the X-linked enzyme glucose-6-phosphate dehydrogenase (G6PD) as a clonal marker. The results were found to depend upon the simultaneous study of tissue from normal mice, mice homozygous for abnormal G6PD levels, and heterozygous mice. A dual population of cells was found in the heterozygous animals in all tissues studied. The oesophageal epithelium showed precisely separated alternating paths of positive and negative cells. Small intestinal crypts were always monophenotypic, whereas villi were polyphenotypic, indicating that crypts are monoclonal and villi derive from more than one

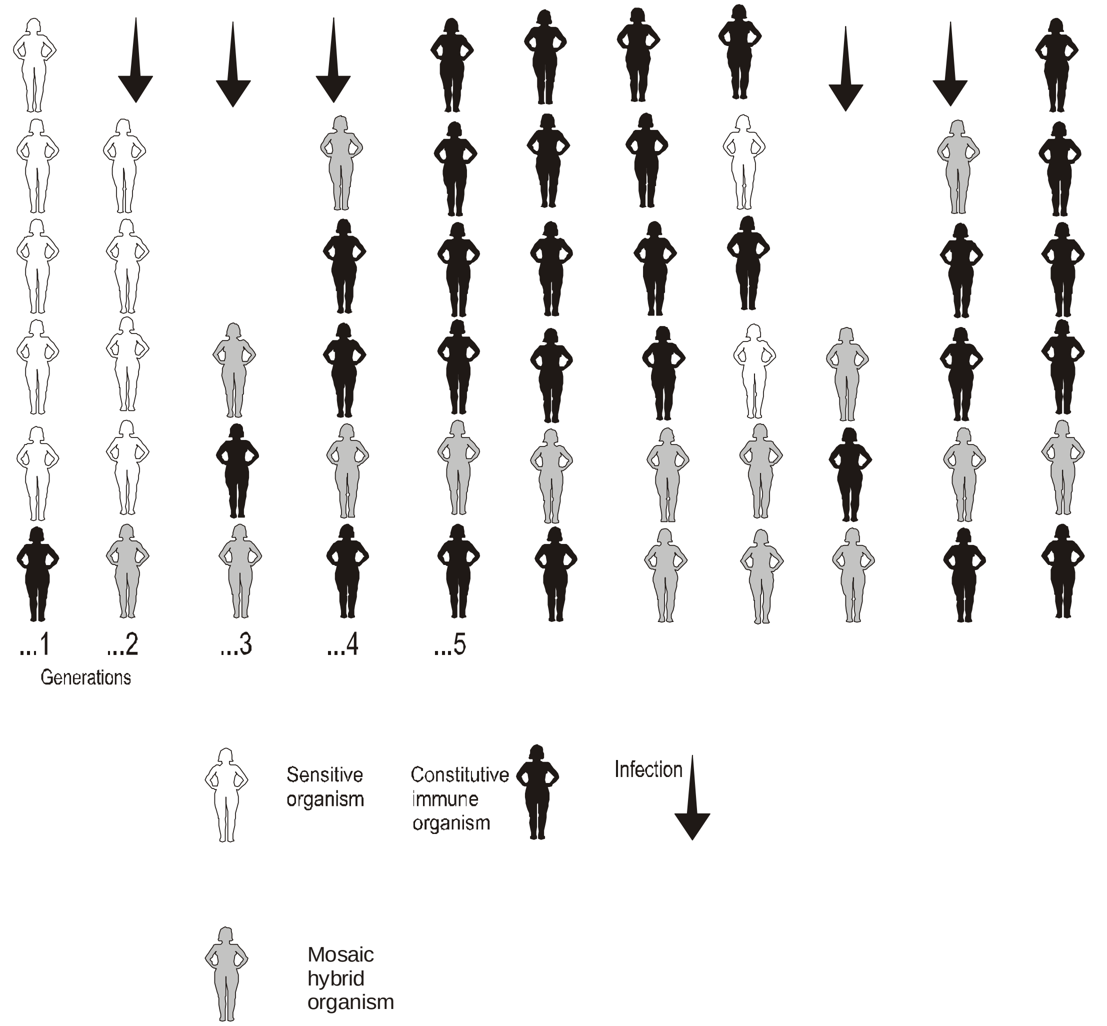

**FIGURE 3.** Transformation of population in the fight against microbes. a, constitutive immune mutant; b, sensitive organism; c, mosaic hybrid organism.

crypt. In contrast, thyroid follicles and breast acini showed a mixture of mono- and polyphenotypia, consistent with a polyclonal origin[34]. Thus, it must not be supposed that all parts of the body are equally liable to specific infectious damage.

In a population reliant on inherent constitutional immunity to an infectious agent, individuals can be conveniently divided into three categories, as follows (Fig. 4):

1. Totally resistant organisms (no susceptible structures)
2. Mildly susceptible organisms in which a few foci appear and the infection runs a benign course
3. Organisms in which the number of susceptible structures is high and the infectious process develops in a severe form with formation of many foci of specific infection[19,21]

## AGE AND IMMUNITY

Constitutional immunity may change during ontogeny of the organism. Immunogenic characteristics of the molecular constitution of the organism are controlled by the genetic program

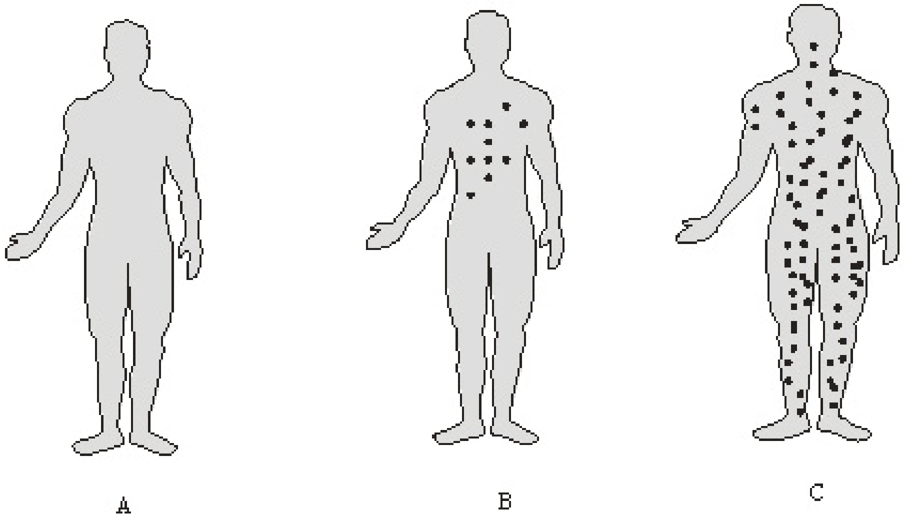

**FIGURE 4.** Individual diversity in the severity of a disease. A, totally resistant organism which contains no susceptible structures; B, mildly susceptible organism in which a few sensitive foci exists and the disease runs a benign course; C, organism in which the number of constitutionally susceptible structures is high and the disease develops in a severe form.

of individual development. That is why newborn and 3-week-old mice are totally defenceless against the virus of yellow fever, but by adulthood they have become completely resistant. Newborn mice are also very susceptible to the herpes virus, but by 4 weeks of age this sensitivity is replaced by constitutional resistance[35,36]. A general characteristic of most infections in animals and plants is the changed response to disease, which develops in the course of growth and maturation. The changes are particularly dramatic in the neonatal period.

Meningococcal infection presents one of the rare exceptions to this common law. About 15% of all cases of childhood meningitis is due to the meningococcus virus[37]; however, children under 1 month of age rarely fall ill with a meningococcal infection. In a series of 211 cases of neonatal meningitis, *Neisseria meningitidis* was an uncommon organism, with only 2 of the 211 due to *N. meningitidis*[38]. In accordance with these epidemic and clinical observations, cells of human newborns are absolutely resistant to the attachment of meningococci[16]. However, in the neonatal and following periods, the cells become far different and individually sensitive to meningococcal adhesion. Thus, the function of meningococcal receptors on human cells develops during postnatal ontogenesis. In contrast, the influenza viral adhesion is characterized by different individual cell sensitivity in all age groups tested[16]. Erythrocytes of 2-day-old chicks are more sensitive to rabies virus adhesion than those of adult chickens[39].

The constitutional immunity of a cell may change during the ontogeny of the cell. Malarial parasites demonstrate selectivity towards a particular age group of erythrocytes. For example, young mature chicken erythrocytes are selectively attacked by *Plasmodium gallinaceum*. Erythrocytes under 12 days of age are preferentially destroyed by the parasites. The factor involved in such a phenomenon may be that the young erythrocytes contain more lipids in their membrane than the older cells[40]. Age-related changes in resistance of cells to mengo virus and encephalomyocarditis are conditioned by corresponding changes in maturation of the cell membrane molecular composition, namely the structure of the molecular receptor[41].

Senescence increases the frequency and severity of infections, which cannot be explained only by the decline in efficiency of the lymphoid system immunogenic response. The increase in susceptibility of the elderly to certain infectious diseases can be explained by the changes in molecular constitution of organs, tissues, cell clones, cells, and extracellular structures that occur with age. There are developmental changes in the molecular constitution of cells that are correlated with developmental differences in their physiological and ecological characteristic, and this may cause the age-related differences in susceptibility to relevant agents.

## PHYSICAL, CHEMICAL, AND HORMONAL INFLUENCES

Physical, chemical, hormonal, nutritional, and other environmental influences may change the strength of constitutional immunity and influence the course and severity of infectious diseases. Penetrating radiation destroys the system of constitutional invulnerability of dogs to anthrax and listeriosis, but the same physical factor does not noticeably influence antiviral constitutional immunity[42]. The lower body temperature of reptiles and fish, compared to mammals, inhibits the action of both tetanic and botulinic toxins. The supercooling of an organism sharply activates the pathogenic character of its interrelations with micro-organisms, which under ordinary conditions are commensal on the mucous membranes.

Certain chemical influences can remake the hereditary features of the molecular constitution of the organism, which forms resistance to microbes. The sugarcane clones susceptible to eyespot disease caused by *Helminthosporium sacchari*, do not show relevant symptoms after pretreatment with one of any number of alpha-galactosides, which inhibit the specific action of toxin[43].

The cells of animals susceptible to the action of the diphtheritic toxin become completely immune to its action after treatment with diethylaminodextran or ammonium chloride. The immunosuppressive effect is obtained after treating the cells with gangliosides[44]. Exogenous GM1 ganglioside has been found to be incorporated into the membrane of exposed cells and thus sensitises the cells to cholera toxin. After treatment of target cells with gangliosides the sensitivity of leucocytes and erythrocytes to adhesion of *N. meningitidis* increases up to 91-fold depending on both the ganglioside concentration and meningococci serogroup[45].

Protection of sensitive mammalian cells from the lethal effect of diphtheria toxin can be provided by treatment of the cells with exogenous nucleotides. Nucleotides were found to antagonize the binding of diphtheria toxin to its cell surface receptors in the manner that correlated with the degree of protection. This protection was not limited to adenine nucleotides. All the common purine and pyrimidine triphosphate nucleotides protected cells to some degree[46].

Nutrition influences constitutional immunity in different ways. All steps of the interaction between parasites and hosts involve the parasites' utilization of the victim's molecular components that determine the nutritional status of the parasite. The infectious process may be considered as competition between parasites and their victim for the biosynthetic products produced by the victim. The good nutrition of a victim is a sufficient predisposition for the success of parasitic aggression. According to generally accepted opinion, however, a healthy, well-fed individual has as much (or more) protection against an infection as that provided by currently available vaccines. However, the malnourished and otherwise compromised victim is considerably more susceptible.

Although malnutrition is traditionally thought to impair host immunity, such an assumption is not always true. In virtually all of the numerous studies reported, malnutrition either contributed to a synergistic increase in the severity of bacterial disease or had no demonstrable effect. The same synergistic trend was evident in most experimental bacterial infections in animals. In contrast, viral infections showed an antagonistic interaction, almost as frequently as a synergistic interaction. Immunity to bacterial infections is usually weaker with a deficit of protein and certain amino acids in food, but resistance to viral infections under the same conditions usually increases[47].

Cell receptivity to several viral and bacterial species fluctuates during the hormonal equilibria characteristic of the menstrual cycle. Oestrogen and progesterone directly affect some biochemical properties of target cells, including the expression of surface components acting as receptors for microbial adhesions. Hormonal imbalances induced by pregnancy are associated with a loss of innate immunity to some viral infections[48]. Hormonal regulation of cell surface receptors has been demonstrated in cells exposed to hormones, neurotransmitters, and many other surface modulating factors. Consequently, the ability of target cells to respond to changes in hormone concentration by regulating the number and affinity of their surface receptors is now widely documented[49].

## CONCLUSIONS

Each infectious disease expresses at least two categories of the same tissue in the infected organism, outwardly identical and differing only in their relationship to a given micro-organism. Some parts of one category are affected by a given microbe, while at the same time morphologically identical components of the organism remain uninvolved although both exist under the same conditions and may be equally attacked by the agent. The origin of those mosaic configurations within affected homogenous structures is determined by genetic factors. Constitutional immunity explains why a given microbe affects parts of one category, and why other morphologically identical components of the same organism are resistant. These genetically predetermined differences are proof of heterozygosity: one of the constitutionally immune parents passes on a lack of receptivity to a certain infection to the descendant in some parts of organs, while the susceptible parts of a different molecular configuration are inherited from the second parent. It can also be by some physical, chemical, or other environmental and physiological influences. In the light of the above conception, the origin of individual differences in a course and severity of infectious diseases is becoming better known. This situation emphasises the importance of further research to determine the respective roles that inbreeding and outbreeding have in the appearance, localization, severity, and clinical course of infectious diseases as well as the new directions of their clinical prognoses and cures.

## ACKNOWLEDGEMENTS

The author respectfully thanks the anonymous referees and Scribendi Inc. for critical reading and helpful correction of this manuscript during its journey to readers.

## BIOSKETCH

**Sergey Rumyantsev**, Ph.D., is Head of the Immunology Department at Andent, Inc., Waukegan, Illinois. His current research interests include: disease prevention and treatment; molecular ecology of microbe-victim ecosystems; mechanisms of constitutional (genetic) immunty in humans, animals, and plants, its molecular bases, evolution, and ontogeny; testing for individual genetic predisposition to infections; molecular genetic bases of humans' structural individuality and their relation to infections' pathogenesis and manifestations; principles of selective vaccinations; contribution of infections to biomolecular evolution; vaccines development; molecular ecology of humans' relationships.
